# Developmental trajectories of physical activity and television viewing during adolescence among girls: National Growth and Health Cohort Study

**DOI:** 10.1186/s12889-015-2043-4

**Published:** 2015-07-15

**Authors:** Soyang Kwon, Jungwha Lee, Mercedes R. Carnethon

**Affiliations:** Department of Pediatrics, Stanley Manne Children’s Research Institute, Ann & Robert H. Lurie Children’s Hospital of Chicago, Northwestern University, 225 E Chicago Ave. Box 157, Chicago, IL 60611 USA; Department of Preventive Medicine, Northwestern University, 680 N Lakeshore Dr. Suite 1400, Chicago, IL 60611 USA

**Keywords:** Latent class growth model, Group-based trajectory model, Dual trajectories, National Growth and Health Study, Physical activity patterns, Television viewing

## Abstract

**Background:**

Analytic methodology for investigating physical activity patterns over time has been limited. The aim of this study was to demonstrate the group-based trajectory analysis process for identifying developmental physical activity (PA) and television (TV) viewing trajectories and the risk factor of PA trajectories, and for examining a relationship between PA and TV viewing trajectories among adolescent girls.

**Methods:**

Secondary analysis was conducted using the National Growth and Health Study (NGHS) dataset. The NGHS administered the Habitual Activity Questionnaire and TV viewing questionnaire to White and Black girls at age 10, 12, 14, 16, 17, 18, and 19 years. Group-based trajectory analyses were conducted to identify distinct PA trajectories. Race was chosen to present an example of the risk factor analysis and was added as a predictor in the trajectory model. Dual-trajectory analysis was conducted to estimate probabilities of TV viewing trajectory groups conditional on the PA trajectory groups.

**Results:**

A total of 2,155 girls (52 % Black) were included in the data analysis. We identified four PA trajectories: substantially decreasing from high PA (PA group 1, 9.4 %), maintaining moderate PA (PA group 2, 31.6 %), maintaining high PA (PA group 3, 5.8 %), and decreasing from moderate PA (PA group 4, 53.2 %). A significantly lower proportion of Black girls had high PA levels at baseline and maintained their baseline PA than White girls. Most girls who were classified as maintaining high PA (88 %) were also classified as decreasing TV viewing.

**Conclusions:**

A group-based trajectory approach provides new insights about the patterns of maintaining moderate or high levels of PA that exist among adolescent girls. However, a lower proportion of Black girls followed the maintenance patterns than White girls. The behavioral development of PA and TV viewing may be intertwined among adolescent girls.

## Background

Habitual moderate- to vigorous-intensity physical activity (MVPA) during childhood provides numerous physical, psycho-social, and cognitive health benefits [1–3]. There is an ongoing international debate regarding the inclusion of sedentary behavior recommendations in the physical activity (PA) guidelines, based on studies of the relationship between PA and sedentary behaviors [4–6] and studies of the independent effect of PA and sedentary behaviors on health outcomes [7–9]. In particular, television (TV) viewing has been repeatedly reported as a discrete sedentary behavior that has been positively associated with obesity and other cardiometabolic risk factors in both boys and girls [9–12]. Longitudinal investigation of PA can provide insights about changes in PA behaviors over time and the relationship of those changes with health outcomes. Nevertheless, capturing diverse patterns of PA over time within a population is a complex analytic issue. Previous studies, which most often summarize longitudinal PA data by calculating the mean of PA change between two time points [13] or use a tracking approach [14], have shown that PA levels substantially decline during adolescence and that PA levels are stable at a low to moderate level during childhood and adolescence [15]. However, these two analytic approaches have drawbacks: they do not allow for potentially different patterns of change in PA levels across individuals, and they are required to hold the assumption that all study participants are drawn from a single homogenous population with common parameters such that all study participants would follow the same PA pattern. Recently, a growth curve model approach has been used to analyze longitudinal PA data from ages 8 to 15 years [7], which allows for fitting inter-individual differences in PA change over time. However, this approach assumes that all individuals in the population follow a similar functional form (e.g., linear, quadratic, cubic) of development [16].

There have also been attempts to identify distinctive patterns of PA change according to subgroups. Using the National Growth and Health Study (NGHS) dataset, Kimm et al. [17] divided girl participants into three groups: active, moderately active, and inactive, based on mean PA levels measured at multiple time points, and showed fairly similar patterns among the three groups, although the absolute level of PA differed by subgroup. Kwon and Janz [18] also divided the members from five cohort studies into three groups based on their baseline PA levels and found that the stability of PA levels over time differed by baseline PA level. However, defining subgroups using prior analysis and subjective classification rules, i.e., by mean PA measured at multiple time points [17], baseline PA [18], or by meeting PA guidelines [19] is fraught with statistical dangers, including the dual risks of creating groups that reflect only random variation and failing to identify important but unusual developmental patterns [20]. Rather than assuming the existence of developmental trajectories of a specific form before performing the statistical analyses, allowing for the hypothesized trajectories to emerge from the data itself will likely produce models that better fit the data [20]. An advanced analytic approach that could both complement traditional analytic approaches and deepen our understanding of the development of PA behavior during childhood and adolescence is needed.

Group-based trajectory modeling is a type of finite mixture model that extends growth curve modeling for distinct subgroups, which allows the shape of the trajectories to vary across groups [20]. Unlike growth mixture modeling, which assumes that a population is composed of literally distinct groups, trajectory modeling uses the trajectory groups as a statistical device for approximating the unknown distribution of trajectories across participants [16] employing a maximum likelihood approach. Thus, this type of group-based trajectory model is useful for identifying meaningful but unknown (or unmeasurable) homogeneous subpopulations (‘trajectory classes’) that follow distinct developmental trajectories of behaviors within a heterogeneous population [21], and for providing an exploratory capacity to identify previously unrecognized developmental patterns.

The group-based trajectory approach has become popular in the field of behavioral and social sciences for studying the developmental trajectories of behaviors [22]. This approach has also begun to be used in recent adult PA studies among general adult populations [23–25] as well as in special populations with medical conditions such as heart disease [26]. In a child study, Janz et al. [27] adopted the group-based trajectory approach to examine bone strength outcomes according to sex-specific developmental trajectories of PA from age 5 to 17 years. More research using the group-based trajectory approach should follow in larger, more diverse, study populations. In this study, we first aimed to demonstrate the group-based trajectory analysis process for identifying distinct developmental PA and TV viewing trajectories. Secondly, we aimed to present an example of risk factor analysis by examining the difference in the distribution of race by PA trajectories. Thirdly, we aimed to examine the interrelationship between PA and TV viewing patterns simultaneously among adolescent girls. The study hypotheses were that the developmental patterns of PA and TV viewing behaviors over adolescence are heterogeneous within the NGHS population and that White girls are more likely to maintain a healthy PA level than Black girls.

## Methods

### Participants

We used the existing NGHS dataset, which was acquired from the Biologic Specimen and Data Repository Information Coordinating Center. NGHS is a 9-year follow-up cohort study that collected data from 1987 to 1997 to determine whether Black-White differences in the development of obesity in pubescent females were due to differences in psychosocial, socioeconomic, and other environmental factors, and whether those differences led to Black-White differences in CVD risk factors [28]. A total of 1,213 non-Hispanic Black girls and 1,166 non-Hispanic White girls at age 9 or 10 years were recruited at three study sites: 887 girls from public and parochial schools in the California Richmond Unified School District, 871 from public and parochial schools in the Cincinnati area, and 621 from the Group Health Association health maintenance organization (HMO) in the Washington DC area. The three areas were chosen based on U.S. census tract data to include a wide distribution of household incomes and parental education levels within each race [28]. All of the 43 elementary schools in the Richmond school district were invited to participate. In the Cincinnati area, 6 traditional public elementary schools, 6 alternative public elementary schools, and parochial elementary schools that “feed” 2 parochial high schools that had partnered with the Cincinnati study center in previous studies were invited. Participants in the Washington DC area were randomly drawn from an HMO membership list. Due to an insufficient sample of White girls from the HMO membership list, a troop of Girl Scouts was additionally recruited from the same geographic area. The eligibility criteria included female, self-declared White or Black race with racially concordant household, age 9 or 10 years within 2 weeks of the first clinic visit, and completion of a socioeconomic survey at baseline (49.0 % Whites; 51.0 % Blacks). The participation rate was 78 %. The follow-up rate was 89 % (91 % for Blacks and 88 % for Whites) at study year 10. Written informed consent was obtained from the parents until the child became 18 years old, when she also gave written consent. The NGHS was approved by University of California-Berkeley Institutional Review Boards (IRB), University of Cincinnati IRB, and Westat IRB. The current study of de-identified existing data was exempt from Ann & Robert H. Lurie Children’s Hospital of Chicago IRB.

### Measurements

The measurements were conducted in the Richmond and Cincinnati participants’ schools and in the Group Health Association clinics for Washington DC participants. Standard protocols were used across all study sites. To ensure the comparability of the data collected at all three locations, a “master trainer” trained and certified local trainers (research staff) who then trained, certified, and monitored all field staff [28]. The habitual activity questionnaire (HAQ), which was adopted from Ku et al. [29] and modified for the NGHS [30], was administered as a structured interview at study years 1, 3, and 5 (participant mean ages 10, 12, and 14 years), and self-administered at study years 7, 8, 9, and 10 (participant mean ages 16, 17, 18, and 19 years). The HAQ asked a girl to list each of the classes/lessons and PA other than sports and classes/lessons (unstructured PA) that she participated in, and to report the frequency and fraction of the year (i.e., most, half, or small part) of her participation in the particular activity. The HAQ is a validated instrument against a 3-day activity diary and accelerometry data to examine longitudinal patterns of PA level over time [30]. The HAQ score, expressed as MET-times per week [30], was computed by multiplying estimated metabolic equivalents (METs; the ratio of metabolic rate during a specific PA to a reference metabolic rate; 1 MET = 3.5 ml O_2_ kg^−1^ min^−1^) for each recorded activity by the weekly frequency (never = 0; less than once a week = 1; 1 or 2 times a week = 2; or ≥ 3 times a week = 3) and the fraction of the year during which it was performed (for classes/lessons: “most” of the year =1, “half” of the year = 0.5, and “small part” of the year = 0.25; for sports and unstructured PA: “most” of the year = 0.75; “half” of the year = 0.375, and “small part” of the year = 0.1875). The duration of activities was not considered because pilot testing revealed that 9 or 10 year-old girls were unable to reliably recall the duration of activities during the previous year [17]. The sum of HAQ scores for all activity categories (continuous variable) was used as an indicator of PA level.

To measure TV viewing behavior at study years 1, 3, and 5, a list of current TV programs was given to participants from which they self-reported the TV programs that they usually watched. The program list was updated once during the year. In addition, girls were asked about the number of hours of TV movies or videos they watched in the past week. Assuming that on average a TV show lasts 0.5 h, TV movies last 1.5 h, and girls watched each reported program for the entire duration [31], weekly hours spent watching TV programs and TV movies and videos were summed as TV viewing time (hours/week). At study years 7, 8, 9, and 10, girls self-reported estimated hours spent watching TV during morning, afternoon, and nighttime hours on each day of a typical week. TV viewing time (hours/week) was calculated by summing seven days of the reported TV viewing hours.

### Statistical analysis

Girls who completed at least four of seven HAQ assessments were included in the data analyses to consider the possibility of quadratic models. Socio-demographic background was compared between those who were excluded from the analyses due to completing less than four PA assessments and those who were included. Descriptive analyses were performed for participant characteristics.

#### Model search

We conducted group-based trajectory analysis in STATA TRAJ [32] to identify subgroups within the NGHS cohort. In the process of determining the number of groups, we initially used a quadratic model for all groups. The final number of groups was determined based on the Bayesian Information Criterion (BIC), trajectory shapes for similarity, and the proportion of cohort members in each class [33]. After identifying the optimal number of groups, the level of the polynomial for each group was reduced until a parameter estimate in the highest function had a p-value less than 0.01. With this final model, each participant was assigned to one of the subgroups based on maximum posterior probability. To label the trajectory groups, we considered a HAQ score between 20 and 40 MET-times per week as moderate PA, 40 MET-times per week or higher as high PA, and 20 MET-times per week or lower as low PA. This model search process was repeated for TV viewing trajectories. To label the TV viewing trajectory groups, we considered TV viewing time between 14 and 28 h per week as moderate TV viewing, 28 h per week or higher as high TV viewing, and 14 h per week or lower as low TV viewing.

#### Model diagnostics

We used four diagnostic measures to judge trajectory model fit, as suggested by Nagin [20]: average posterior probability of assignment for each group is 0.7 or higher; odds of correct classification are 5.0 or higher; the proportion of a sample assigned to a certain group is close to the proportion estimated from the model; and 99 % confidence intervals of the estimated proportion are reasonably narrow.

#### Risk factor analysis

We chose race to present an example of risk factor analysis in a trajectory model. Race was chosen because the primary purpose of the NGHS was to compare Black and White adolescents in the development of obesity. To examine the difference in the distribution of race by PA trajectories, we included the race variable (reference group: White) as a predictor in the final group-based trajectory model [34].

### Dual trajectory analysis

To investigate the relationship between the development of PA and TV viewing behaviors during adolescence among girls, we conducted a dual trajectory model that summarizes the dynamic interrelationship between two longitudinal variables across various trajectory groups, instead of a traditional association analysis that estimates the overall association between two variables over heterogeneous subpopulations. All models, including the dual-trajectory model, converged and all parameters had reasonably small standard errors (all of the standard errors divided by the means were less than 0.3). Therefore, starting values were not specified and default starting values were used.

## Results

The proportion of non-missing data for PA was 96 % at study year 1, 92 % at year 3, 85 % at year 5, 79 % at year 7, 82 % at year 8, 83 % at year 9, and 85 % at year 10. Of the 2,379 NGHS participants, 2,155 girls who had at least four PA assessments (89 % of Whites and 92 % of Blacks) were included in the PA data analysis. Those who were included in the analysis were less likely to have lower parental education and household income, compared with those who were excluded from the analysis (*n* = 224): ≤ high school education = 25 % vs. 38 % (*P*-value < 0.05); annual income of < $10,000 = 17 % vs. 25 % (*P*-value < 0.05).

Of those who were included in the data analysis, the percentage of parental education ≤ high school was 19 % for White girls and 30 % for Black girls (Table 1). At baseline, the percentage of overweight was 7 % among Whites and 17 % among Blacks. Sixty-two percent of participants completed all seven PA assessments, 22 % completed six assessments, 10 % completed five assessments, and 6 % completed four assessments. The mean of PA levels at each assessment was higher among White girls than Black girls.

**Table 1:** Descriptive analysis. National Growth and Health Study

	White girls (*n* = 1,036)	Black girls (*n* = 1,119)	*P*-value
Parental education level, n (%)			
≤ High school	194 (18.7 %)	339 (30.3 %)	
Some college	320 (30.9 %)	540 (48.3 %)	<0.01
≥4-year college	521 (50.3 %)	239 (21.4 %)	
HAQ scores (MET-times/wk), mean ± SD			
Age 10 years	33.3 ± 18.9	30.4 ± 19.5	<0.01
Age 12 years	25.5 ± 15.7	23.1 ± 15.8	<0.01
Age 14 years	22.7 ± 15.9	17.4 ± 13.5	<0.01
Age 16 years	13.9 ± 14.3	6.1 ± 10.1	<0.01
Age 17 years	11.7 ± 14.0	5.4 ± 10.3	<0.01
Age 18 years	15.6 ± 15.7	7.0 ± 11.8	<0.01
Age 19 years	17.9 ± 19.8	7.2 ± 13.0	<0.01
TV viewing hours (hours/wk), mean ± SD			
Age 10 years	24.9 ± 14.1	35.9 ± 17.0	<0.01
Age 12 years	27.4 ± 15.8	45.8 ± 16.6	<0.01
Age 14 years	24.9 ± 14.7	41.6 ± 16.8	<0.01
Age 16 years	19.6 ± 14.3	39.1 ± 18.9	<0.01
Age 17 years	17.9 ± 14.8	36.6 ± 19.7	<0.01
Age 18 years	17.4 ± 15.4	36.3 ± 20.3	<0.01
Age 19 years	18.9 ± 16.6	38.9 ± 20.2	<0.01

*SD* standard deviation

We identified four distinct PA trajectories over a 9-year period from age 10 to 19 years (Table 2). In determining the number of PA trajectory groups, although the BIC was slightly higher for the five group model (BIC = -47,948) than for the four group model (BIC = -48,014), we chose four groups because the difference in BIC was small, and because one additional group resulted from splitting the largest proportion group that did not show distinctive patterns, which did not help to further our understanding of the development of PA behavior. Of the four group trajectories, one trajectory was a quadratic model, two were linear models, and the other was a constant model (Table 2). The four group model was judged to be adequate based on the four model diagnostic criteria (Table 3).

**Table 2:** Model search process for physical activity (PA) trajectories

Determining the number of PA trajectory groups								
Number of groups			BIC			Smallest group %		
2			−48,370			29.0		
3			−48,182			6.1		
4			−48,014			5.6		
5			−47,948			3.0		
			↓					
The final number of PA trajectory groups was determined to be 4.								
			↓					
Determining the highest model function of the 4 PA trajectory groups								
	1^st^ iteration		2^nd^ iteration		3^rd^ iteration		4^th^ iteration	
Group	Highest function	*P-value*	Highest function	*P-value*	Highest function	*P-value*	Highest function	*P-value*
Group 1	Quadratic	<0.001	Quadratic	<0.001	Quadratic	<0.001	Quadratic	<0.001
Group 2	Quadratic	<0.001	Quadratic	<0.001	Quadratic	0.14	Linear	<0.001
Group 3	Quadratic	<0.001	Quadratic	0.27	Linear	0.43	Constant	<0.001
Group 4	Quadratic	0.72	Linear	<0.001	Linear	<0.001	Linear	<0.001

*BIC* Bayesian information criterion

**Table 3:** Diagnostics of assignment accuracy

Group	Estimated proportion from the trajectory model (π)	99 % confidence interval for π	Proportion by posterior probability-based classification	Average posterior probability	Odds of correct classification
Group 1	9.4 %	9.1, 9.6	8.0 %	81.2 %	42.8
Group 2	31.6 %	31.5, 31.8	30.9 %	83.1 %	10.7
Group 3	5.8 %	5.6, 6.0	5.4 %	88.2 %	121.3
Group 4	53.2 %	53.0, 53.3	55.7 %	89.2 %	7.2

**Fig. 1:**
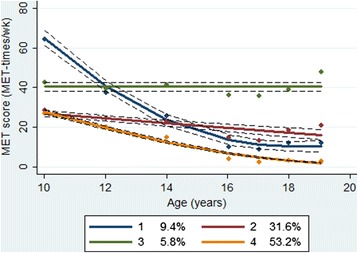
Mean Habitual Activity Questionnaire scores and 95 % confidence intervals by PA trajectory classes. Dots indicate actual mean Habitual Activity Questionnaire (HAQ) scores, a solid line indicates estimated mean HAQ scores, and a dotted line indicates 95 % confidence intervals of estimated mean HAQ scores. MET, metabolic equivalent

Based on the graphical presentation (Fig. 1), the four groups were labeled as ‘substantially decreasing from high PA’ (PA group 1, 9.4 %), ‘maintaining moderate PA’ (PA group 2, 31.6 %), ‘maintaining high PA’ (PA group 3, 5.8 %), and ‘decreasing from moderate PA’ (PA group 4, 53.2 %). PA groups 1 and 4 (62.6 %) showed a declining pattern while PA groups 2 and 3 (37.4 %) showed a maintaining pattern. PA groups 1 and 3 (15.2 %) had high PA levels at baseline, and PA groups 2 and 4 (84.8 %) had moderate PA levels at baseline. Among Whites, 8 % belonged to PA group 1, 43 % to group 2, 10 % to group 3, and 39 % to group 4. Among Blacks, 8 % belonged to PA group 1, 20 % to group 2, 1 % to group 3, and 71 % to group 4. Black girls were less likely to be in the maintaining high PA group than White girls (*P*-value < 0.05).

**Fig. 2:**
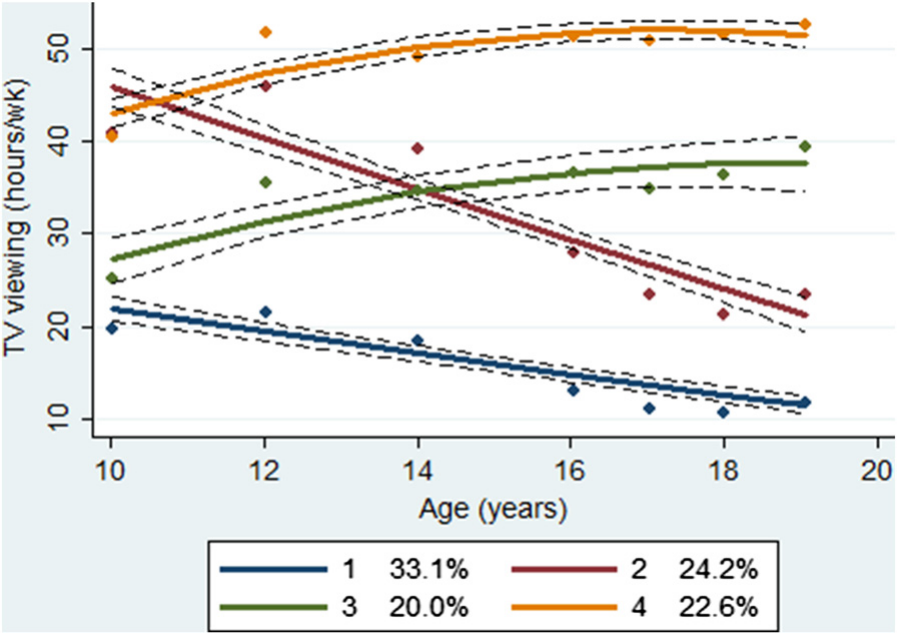
Mean television viewing hours and 95 % confidence intervals by television viewing trajectory classes. Dots indicate actual mean television viewing hours, a solid line indicates estimated mean television viewing hours, and a dotted line indicates 95 % confidence intervals of estimated mean television viewing hours

The proportion of non-missing data for TV viewing was 96.1 % at study year 1, 90.1 % at year 3, 83.9 % at year 5, 78.4 % at year 7, 81.8 % at year 8, 83.1 % at year 9, and 84.7 % at year 10. Of the 2,155 girls who completed at least four PA assessments, 2,150 completed at least four TV viewing assessments and were included in the dual-trajectory analysis. There was no participant who had at least four TV viewing assessments and less than four PA assessments. We identified four TV viewing trajectories, including two linear trajectories and two quadratic trajectories through the model search process. The models were judged to be adequate based on the model diagnostics. We labeled the four groups as follows: ‘decreasing from moderate TV viewing’ (TV group 1, 33.1 %); ‘decreasing from high TV viewing’ (TV group 2, 24.2 %); ‘increasing from moderate TV viewing’ (TV group 3, 20.0 %); and ‘increasing from high TV viewing’ (TV group 4, 22.6 %) (Fig. 2). Among Whites, 61 % belonged to TV group 1, 20 % to group 2, 14 % to group 3, and 5 % to group 4. Among Blacks, 9 % belonged to TV group 1, 28 % to group 2, 24 % to group 3, and 38 % to group 4. Black girls were less likely to follow the healthiest TV viewing pattern (TV group 1; *P*-value < 0.05). Probabilities of TV viewing trajectory groups conditional on PA trajectory groups are presented in Table 4. Most girls who were classified as maintaining high PA (88 % of PA group 3) were also classified as decreasing TV viewing (TV groups 1 and 2). Conversely, most girls who represented the unhealthiest PA pattern (86 % of PA group 4) followed an increasing pattern of TV viewing (TV groups 3 and 4).

**Table 4:** Probabilities of TV viewing trajectory groups conditional on PA trajectory groups. National Growth and Health Study

	TV Group 1	TV Group 2	TV Group 3	TV Group 4	Total
			%		
All PA groups	10.4	27.0	35.3	27.3	100
PA group 1	1.6	29.8	43.0	27.2	100
PA group 2	17.3	39.5	31.7	11.4	100
PA group 3	45.6	42.1	7.5	4.8	100
PA group 4	0.0	13.8	42.1	44.0	100

Note. *PA* physical activity, *TV* television

The probabilities above were estimated from a dual-trajectory analysis of physical activity and television viewing behaviors

PA group label: 1 = substantially decreasing from high PA; 2 = maintaining moderate PA; 3 = maintaining high PA; 4 = decreasing from moderate PA

TV group label: 1 = decreasing from moderate TV viewing; 2 = decreasing from high TV viewing; 3 = increasing from moderate TV viewing; 4 = increasing from high TV viewing

## Discussion

Our group-based trajectory analysis offers the following new insights for understanding PA patterns among adolescent girls. First, we identified four distinct PA development trajectories, including two maintenance patterns (maintaining high PA and maintaining moderate PA) and two declining patterns. One in three girls followed the two maintenance patterns. However, a lower proportion of Black girls followed maintenance trajectories than White girls. Second, this study suggests that the developmental patterns of PA and TV viewing behaviors may cluster.

The current study results are consistent with a group-based trajectory analysis by Kwon et al. [35] which also identified a consistently active pattern in the Iowa Bone Development Study (IBDS) cohort. However, the study by Kwon included both boys and girls together in the analysis. An earlier study by Janz et al. [27] reported three PA trajectories for girls from age 5 to 17 years in the IBDS cohort, all of which showed a declining trend over time and reached a similar level of PA at age 17. The conflicting results from the Janz study can be partly attributed to its relatively small sample size (*n* = 263), where a consistently active trajectory, which would have represented only a small number of the study participants, may not have been detectable. Future research should confirm the existence of a consistently active pattern among girls in large cohort studies.

Our finding of an unhealthy PA pattern among Black girls is not surprising. Previous studies have consistently reported lower PA levels among Black girls compared to White girls [36, 37]. The finding may imply that Black girls are less likely to experience individual and environmental factors that promote PA, such as self-efficacy, perceived behavioral control, land-use mix, and residential density [38]. Our findings regarding a relationship between the behavioral development of PA and TV viewing also provide a different perspective from previous observational studies [4, 6]. Previous studies have reported no association between PA and sedentary behaviors, which support that TV viewing and PA behaviors are separate constructs in boys and girls [39]. However, the present study suggests that the behavioral development of PA and TV viewing during adolescence among girls may be intertwined. Most girls (86.1 %) who presented the most common PA pattern of decreasing from moderate PA concurrently developed an increasing pattern of TV viewing. Conversely, most girls (87.7 %) with the healthiest PA pattern of maintaining high PA concurrently developed a decreasing pattern of TV viewing. Our findings should be confirmed in subsequent dual trajectory analyses using existing longitudinal study datasets such as the IBDS and the National Longitudinal Study of Adolescent Health.

We note a few cautions in conducting and interpreting group-based trajectory analysis. First, the group-based trajectory approach is not universally appropriate for longitudinal PA data. Rather, an appropriate analytic approach should be chosen based on the research question at hand. The group-based trajectory approach will be most useful for examining different PA patterns among a heterogeneous population. In addition, the group-based trajectory approach that identifies groups of individuals who share particular attributes (also called a person-centered approach) is more translational to identifying and characterizing subpopulations, as opposed to a variable-centered approach that describes associations between variables [40]. Second, group-based trajectory models, in particular dual-trajectory models and risk factor models, are occasionally not converted and require the specification of starting values. All trajectory models that were fit in this study were converted without specifying starting values. To conduct group-based trajectory analysis, one should pay close attention to the size of standard errors and ensure that the model has reached the maximum ability to obtain estimates that are closest to the truth. Third, because group membership was determined based on the maximum likelihood, not all group members perfectly followed their group’s trajectory. In this study, we assumed that individuals within a group were homogenous, and we did not specify random effects in the trajectory models to simplify and avoid over-adjusting. Within-heterogeneity can be estimated and each trajectory can be further validated [41]. Group membership may change as variation (covariates) is added and group members become more heterogeneous.

### Limitations

Several limitations of our study should be acknowledged. First, the observations began at age 10 years, which limits our understanding of the development of PA behaviors at earlier ages when a low level of PA is known to be established [42]. However, the significance of this study lies in showing PA development during adolescence when substantial changes in PA behaviors are known to occur. Second, self-reported PA and TV viewing assessments are prone to measurement error. In particular, changes to the items on the TV viewing questionnaire (from checking off TV shows watched from the TV show list to reporting the total amount of TV viewing hours by the segment of the day) might have resulted in a differential bias in estimating total daily TV viewing hours. Third, this study used relatively older data, which has limited relevance for today’s adolescent population. Also, because the sample was not representative beyond the source population, the study results may not be generalizable to broader populations. Fourth, because a group-based trajectory model assumes that missing data is random, nonrandom missing data over time could have biased the study findings. However, of those who were included in the data analysis, the majority (62 %) completed all seven PA assessments and another 22 % completed six PA assessments. Furthermore, the average number of completed assessments did not differ by race or parental education levels. Therefore, we believe that it is unlikely that missing data for those included in the data analysis would have affected the study findings. However, it should be noted that those who were excluded from the data analysis (5.8 % of the NGHS participants) had a lower socioeconomic background and, therefore, might have had different distributions in PA trajectories. Lastly, although we used a currently available and accepted method to determine the number of trajectory classes, the merits of this method are still up for debate.

## Conclusions

We identified four distinct PA trajectories in adolescence among Black and White girls. One in three girls followed trajectories that maintained their baseline PA levels. However, the proportion of Black girls who followed maintenance trajectories was lower than for White girls. The behavioral development of PA and TV viewing may be intertwined in adolescence among girls, which has long-term implications for cardiovascular risk in adulthood.

## Abbreviations

HAQ: Habitual activity questionnaire
MVPA: Moderate- to vigorous-intensity physical activity
NGHA: National Growth and Health Study
PA: Physical activity
TV: Television
